# Retraction Note: RNA interferences targeting the Fanconi anemia/BRCA pathway upstream genes reverse cisplatin resistance in drug-resistant lung cancer cells

**DOI:** 10.1186/s12929-026-01232-3

**Published:** 2026-03-11

**Authors:** Chun-Hua Dai, Jian Li, Ping Chen, He-Guo Jiang, Ming Wu, Yong-Chang Chen

**Affiliations:** 1 Department of Radiation Oncology, Affiliated Hospital of Jiangsu University, Zhengjiang, 212001 China; 2 Department of Pulmonary Medicine, Affiliated Hospital of Jiangsu University, Zhengjiang, 212001 China; 3 Institute of Medical Science, Jiangsu University, Zhengjiang, 212013 China

**Retraction: Journal of Biomedical Science (2015) 22:77** 10.1186/s12929-015-0185-4

The Editor-in-Chief retracted this article because of concerns regarding a number of figures presented in this work. These concerns call into question the article's overall scientific soundness. An investigation conducted after its publication discovered similarities between images in this work and between images in this work and images in [1],

- The *Beta-actin* bands in Figs. 1B and 1C appear to overlap;
- Portions of the *Beta-actin* bands in Figs. 3A and 3C appear to overlap after a rotation;
- Several bands in Figs. 8A and 8B appear to overlap with one another;
- The *FANCF* band in Fig. 3A appears to overlap, after a rotation, with the *p-Chk2/MCF-7* band in Fig. 8a in [1];
- The *FANCF* band in Fig. 3B appears to overlap, after a rotation, with the *p-ATM/MCF-7* band in Fig. 8a in [1];
- The *Beta-actin* band in Fig. 3B appears to overlap with the *Beta-actin/MDA-MB-231* band in Fig. 8a in [1].

In addition, concerns were raised regarding the authenticity of the statistical data reported in Figs. 2, 3, 7, and 8. The Editor-in-Chief therefore no longer has confidence in the integrity of the research presented in this article.

The authors agree with this retraction.
